# Real-World-Feasible Immunohistochemistry of ATRX, DAXX, and Menin Identifies a Subgroup of Non-Functioning Pancreatic Neuroendocrine Tumors with low Recurrence Risk to Guide De-Escalating Surveillance

**DOI:** 10.1007/s12022-026-09933-z

**Published:** 2026-08-29

**Authors:** Anna Vera Ditte Verschuur, Wenzel Maximillian Hackeng, Jasvir Jairam, Busra Eldem, Jasmijn Sterre Westendorp, Menno Reginaldus Vriens, Gerlof Dirk Valk, Antonio Pea, Roberto Salvia, Rita Teresa Lawlor, Aldo Scarpa, Claudio Luchini, Christopher Michael Heaphy, Seung-Mo Hong, Ilaria Marinoni, Aurel Perren, Koen Marie Anton Dreijerink, Elisabeth Jacqueline Maria Nieveen van Dijkum, Arantza Fariña Sarasqueta, Aatur Dilip Singhi, Sjoerd Geert Elias, Lodewijk Adriaan Anton Brosens

**Affiliations:** 1 Department of Pathology, University Medical Center Utrecht, Utrecht University, Heidelberglaan 100, 3584 CX Utrecht, The Netherlands; 2 Department of Endocrine Surgery, University Medical Center Utrecht, Utrecht, Netherlands; 3 Department of Endocrine Oncology, University Medical Center Utrecht, Utrecht, Netherlands; 4 Department of Pancreatic Surgery, University of Verona, Verona, Italy; 5 ARC-Net Research Center, Department of Engineering for Innovation Medicine, University of Verona, Verona, Italy; 6 ARC-Net Research Center, Department of Diagnostics and Public Health, Section of Pathology, University of Verona, Verona, Italy; 7 Section of Hematology and Medical Oncology, Boston University School of Medicine and Boston Medical Center, MA Boston, United States; 8 Department of Pathology, Asan Medical Center, University of Ulsan College of Medicine, Seoul, Republic of Korea; 9 Institute of Tissue Medicine and Pathology, University of Bern, Bern, 3008 Switzerland; 10 Department of Endocrinology, Amsterdam University Medical Centers, location VU University, Amsterdam, The Netherlands; 11 Department of Endocrine Surgery, Amsterdam University Medical Centers, Amsterdam, The Netherlands; 12 Cancer Center Amsterdam, Department of Pathology, Amsterdam University Medical Centers, Location University of Amsterdam, Amsterdam, the Netherlands; 13 Department of Pathology, University of Pittsburgh Medical Center, Pittsburgh, PA USA; 14 Department of Epidemiology, Julius Center for Health Sciences and Primary Care, UMCU, Utrecht University, Utrecht, the Netherlands

**Keywords:** Neuroendocrine tumors, Biomarkers, ATRX, DAXX, Menin, Immunohistochemistry, Pancreas

## Abstract

Non-functioning pancreatic neuroendocrine tumors (NF-pNETs) show a variable prognosis. Despite 40–60% of patients remaining recurrence-free after surgery, guidelines recommend ≥ 10 years of follow-up. Recent studies identify prognostic subgroups based on ATRX, DAXX, and MEN1 mutations and chromosomal aneuploidy, highlighting a subgroup with favorable prognosis. We aimed to classify resected NF-pNETs into molecular subgroups using ATRX, DAXX, and Menin immunohistochemistry as surrogate markers for genomic status. To do so, we retrospectively collected resected primary sporadic grade 1 and 2 NF-pNETs without synchronous metastases from five international local pathology archives. ATRX, DAXX, and Menin immunohistochemistry was performed, and tumors were categorized into three groups: ATRX-DAXX-loss-group (ATRX or DAXX loss), isolated-Menin-loss-group (Menin loss without ATRX and DAXX loss), and unspecified-group (retained Menin, ATRX and DAXX). Kaplan-Meier analysis evaluated prognostic differences, and multivariable Cox regression assessed the added value of molecular subgroups beyond established prognostic factors. In total, 139 patients with grade 1 (64%) and 2 (36%) NF-pNETs were included. The median follow-up was 28 months (range 0–195) and recurrences occurred in 20% (28/139). No recurrences occurred in the isolated-Menin-loss-group (0/33), versus 12.5% (8/64) in the unspecified-group and 48% (20/42) in the ATRX-DAXX-loss-group. Kaplan-Meier analysis showed better recurrence-free interval in the isolated-Menin-loss-group than other subgroups (*P* < 0.001). In univariate analysis, the isolated-Menin-loss-group had a lower recurrence hazard (HR 0.03; 95%CI: 0.00–0.55; *P* = 0.018), than other subgroups, with little change in the hazard ratio after adjustment, but loss of significance. In conclusion, molecular subclassification by immunohistochemistry identifies NF-pNET patients with a low risk of recurrence and lays the groundwork for future prospective studies assessing genetics-based risk stratification as a tool for guiding clinical management.

## Introduction

Pancreatic neuroendocrine tumors (pNETs) are the second most common neoplasms in the pancreas. Although some pNETs can cause symptomatic hormone overproduction, the vast majority lack functional hormone production and are called non-functioning pNETs (NF-pNETs). NF-pNETs are often identified incidentally and their incidence is rising [1, 2]. This increase is primarily owed to the increased quantity and improved quality of abdominal imaging. While active surveillance is considered for small NF-pNETs (≤ 2 cm), larger tumors are typically resected [3, 4]. NF-pNETs have a variable prognosis [3–6], approximately 40–60% of patients do not recur after resection and are thus cured [7]. Nevertheless, international guidelines recommend a minimum of 10 years of follow-up, often lifelong, including somatostatin receptor (SST)-PET-CT scans every 6–12 months for 5 years, every 12–24 months for the next 5, and every 5 years thereafter [3–6]. These procedures entail substantial healthcare costs and may contribute to patient (sc)anxiety, thereby impacting quality of life.

Although several histopathological and molecular characteristics including larger tumor size, higher tumor grade, presence of lymph node metastases, perineural invasion, ATRX or DAXX loss, and the presence of alternative lengthening of telomeres (ALT), have been associated with risk of recurrence and metastatic disease [8–16], none of these factors are currently used to personalize post-operative follow-up of patients who underwent resection of an NF-pNET. Recent studies have identified prognostic molecular subgroups based on *ATRX*,* DAXX*, *MEN1/*chromosome 11 genetic status [17, 18]. Of interest, one subgroup, defined by isolated *MEN1* mutations and/or chromosome 11 loss but without *ATRX* and *DAXX* loss or other copy number variations (CNVs), seems to have a very low risk of recurrence. The other two subgroups, defined by either presence of *ATRX* or *DAXX* mutations typically with a characteristic pattern of copy number losses, or variable aneuploidies and the absence of *ATRX* and *DAXX* mutations, showed higher risk of metastasis or disease recurrence [17, 18]. Hence, we hypothesize that patients with a tumor with isolated Menin loss may not need long term post-operative follow-up.

Mutation and copy number analyses are costly, time-consuming and not world-wide available. Instead, inactivation of *ATRX*,* DAXX*,* and MEN1* can be readily and affordably assessed using immunohistochemistry for their corresponding proteins. In this multicenter, international retrospective cohort study we investigated whether similar prognostic molecular subgroups could be identified using ATRX, DAXX, and Menin immunohistochemistry as surrogate for molecular analysis [19, 20]. In addition, we investigated the prognostic significance of these immunohistochemistry based subgroups in relation to other established histopathological prognostic factors. 

## Methods

### Patients and Samples

The surgical pathology archives of the University Medical Center Utrecht (UMCU, *n* = 52), Amsterdam University Medical Centers (AUMC, *n* = 21), Asan Medical Center (AMC, *n* = 8), University of Pittsburgh Medical Center (UPMC, *n* = 51) and Bern University (BU, *n* = 17) where searched to identify patients who underwent resection of NF-pNETs between 1993 and 2023. Tumor tissue samples were collected and clinicopathologic and follow-up data, including sex, age, tumor size, tumor location, 2022 WHO grade, Ki-67 proliferation index, regional node (pN) stage, primary tumor (pT) stage, perineural invasion (PNI), lymphovascular invasion (LVI) were obtained from medical records. As the WHO 2026 classification subdivides G2 tumors into G2a and G2b categories based on the Ki-67 proliferation index, the 2026 WHO grade was calculated for cases with available Ki-67 data [21]. Recurrence-free interval (RFI) was calculated from the date of primary tumor resection to the first documented local recurrence in the pancreas, and/or new regional lymph nodes localization, and/or the development of distant metastases and censored at the date of last follow-up. Patients were included if they had a sporadic WHO grade 1 (G1) or grade 2 (G2) NF-pNET, according to the 2022 WHO classification [22], without synchronous metastases at time of primary resection. Patients with functioning tumors or with a hereditary syndrome such as multiple endocrine neoplasia type 1 (MEN1), von Hippel-Lindau (VHL), neurofibromatosis type 1 (NF1) and tuberous sclerosis complex (TSC) syndromes, and perioperative mortality cases were excluded. Cases with insufficient material for ancillary studies or missing data on recurrence-free interval were excluded. The cohort included 49 NF-pNETs of a larger NF-pNET cohort in which ATRX immunohistochemistry, DAXX immunohistochemistry and ALT in relation to prognosis were studied [8]. The study was approved by the local Biobank Research Ethics Committees according to Dutch law and regulations (University Medical Center Utrecht Biobank Research Ethics Committee TcBio approval number #17–881 and # 21–507). Participants were informed about the potential use of their materials and data for research purposes and had the opportunity to object; individuals who objected were excluded from the study. 

### ATRX, DAXX, Menin Immunohistochemistry

Tumor tissue samples were arranged in triplicate from the same tissue block onto a tissue microarray (TMA), as previously described [23]. TMAs and if inconclusive, whole slides were used for ATRX, DAXX and Menin immunohistochemistry and telomere-specific fluorescence in situ hybridization (FISH).

4µm slides of paraffin-embedded tissue were cut and stained using a Ventana Benchmark Ultra *(Roche Diagnostics*,* Almere*,* The Netherlands)*. DAXX (*Atlas Antibodies*,* Bromma*,* Sweden*,* HPA008736)* was detected using CC1 24’ as pre-treatment, the antibody was incubated for 32 min with a 1:75 dilution. ATRX (*Sigma*,* St. Louis*,* USA*,* MO*,* HPA0001906)* was detected using CC1 24’ as pre-treatment, the antibody was incubated for 32 min with a 1:200 dilution. Menin *(Bethyl Laboratories*,* Massachusetts*,* USA*,* IHC00572)* was detected using CC1 32’, the antibody was incubated for 60 min (1:200) including amplification.

ATRX, DAXX, and Menin immunohistochemistry was independently assessed by two reviewers from a group of four investigators (WH, AV, JJ and BE) who were blinded to the clinical data and each other’s results. Any disagreements in scoring were resolved through consensus with an experienced pathologist (LAAB). ATRX, DAXX, and Menin protein were considered “retained” if by immunohistochemistry neoplastic cells showed nuclear staining. Loss of staining was defined as the complete absence of nuclear staining in neoplastic cells, provided there was an adequate internal control (e.g., nuclear labeling of adjacent endothelial cells, lymphocytes, or islets of Langerhans). In the absence of an internal control, the case was considered not interpretable. In such cases, the whole slide of the tumor was re-stained and scored similarly. If the whole slide was non-informative, and together with other stainings (ATRX/DAXX/Menin) or FISH (ALT) the case could not be subjected to one of the three molecular subgroups, the case was excluded from analysis. This resulted in the exclusion of 10 cases: one due to non-informative DAXX staining, one due to non-informative DAXX and Menin staining, six due to non-informative Menin staining, one due to non-informative ATRX staining, and one due to non-informative ATRX and Menin staining. 

### Fluorescence In Situ Hybridization

ALT had previously been evaluated in a subset of cases [8]. ALT was detected using telomere-specific FISH with a fluorescently labeled telomeric-C PNA probe and was defined by the presence of large, ultrabright intranuclear telomere FISH signals in at least 1% of tumor nuclei, with individual foci exhibiting signal intensities more than tenfold higher than those observed in stromal cells [8, 20]. 

### Definition of Molecular Subgroups and Study Outcomes

**Fig. 1:**
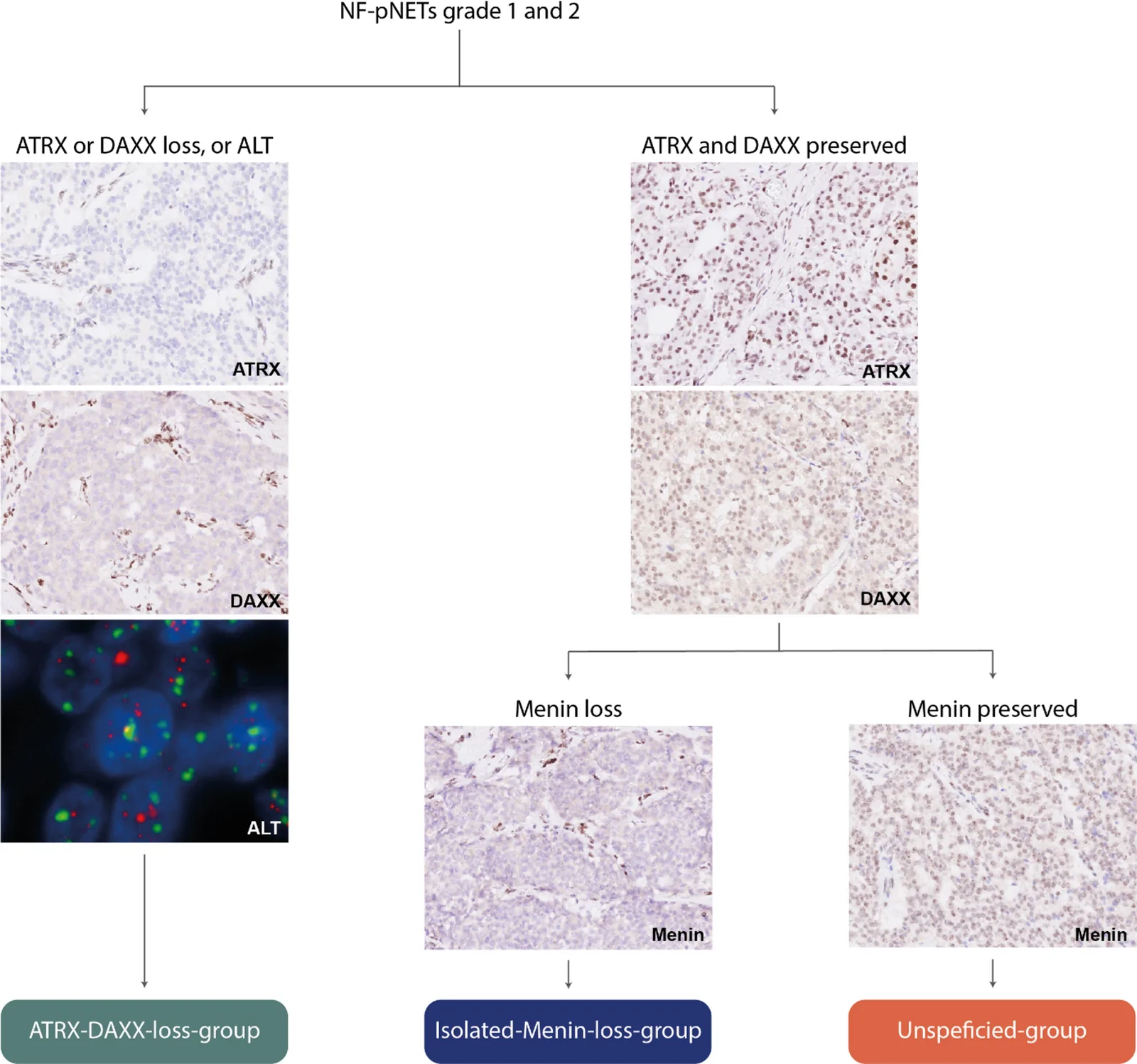
Schematical visualization of the definition of molecular pancreatic neuroendocrine tumor subgroups. ATRX-DAXX-loss-group with ATRX or DAXX loss and/or presence of alternative lengthening of telomeres (ALT) with or without Menin loss, isolated-Menin-loss-group with Menin loss, and preserved ATRX and DAXX expression and absence of ALT, and unspecified-group with both preserved ATRX and DAXX and absence of ALT and preserved Menin

Based on immunohistochemistry and telomere FISH the following three mutually exclusive molecular subgroups were established for analysis: (1) ATRX-DAXX-loss-group with tumors with ATRX or DAXX loss and/or presence of ALT with or without Menin loss; (2) isolated-Menin-loss-group with tumors with Menin loss, and preserved ATRX and DAXX expression and absence of ALT; (3) unspecified-group with tumors that have preserved Menin, ATRX and DAXX expression, absence of ALT. The groups are schematically visualized in Fig. 1. The primary outcome was RFI across the three molecular subgroups. Secondary outcomes included the distribution of clinical and pathological variables between the three subgroups. In addition, recurrence free interval analyses were performed and multivariable Cox regression was used to explore whether the three molecular subgroups have independent prognostic value relative to established prognostic factors (i.e. WHO grading, lymph node metastasis, and perineural invasion). 

### Statistical Analyses

Statistical analyses were performed using R (version 4.5.1), utilizing packages ‘*survival*’ (version 3.8-3), ‘*bpcp*‘(version 1.5.1), ‘*coxphf*’ (version 1.13.4) and ‘*smcfcs*’ (version 2.0.1), and ‘*mice*’ (version 3.18.0). Based on the distribution, the data were described with mean and standard deviation (SD) or median and interquartile range (IQR). For categorical data, the number and proportion (%) were displayed. For comparisons between the three molecular subgroups, categorical variables were assessed using the χ² test or the Fisher–Freeman–Halton exact test, and continuous variables were analyzed using the ANOVA or Kruskal–Wallis tests depending on data distribution. Recurrence-free interval curves were generated using the Kaplan–Meier method, and differences between groups were evaluated using the log-rank test. One-, five-, and ten-year recurrence-free interval survival probabilities were estimated using the *survival* package. When 95% confidence intervals could not be obtained, the Beta Product Confidence Procedure, implemented in the *bpcp* package, was applied.

To assess the prognostic value of the three molecular subgroups relative to the linear predictor for pNET RFI described by Genç et al. [10], we constructed a univariable Cox regression analysis of the molecular subgroups, and a multivariable model including both the linear predictor and the molecular subgroups. We used multivariable Cox regression with Firth correction in view of the small sample size [24]. Due to missing data for several clinicopathologic variables (Table 1), we performed multiple imputation prior to analysis. This was conducted using the R package “smcfcs” with a cox regression for recurrence-free interval as the substantive model, incorporating five variables (2022 WHO grade, PNI, N stage, Menin and variable combining ATRX, DAXX and ALT). Because at least one missing value among these five variables was present in 46% of the patients, 54 imputed datasets were generated, with 25 iterations performed. Cases were assigned to a subgroup based on immunohistochemical results and Rubin’s rules were used to pool results across imputed datasets. For all statistical analysis, a *p*-value of < 0.05 was considered statistically significant.

**Table 1:** Clinical and pathological comparison of three molecular subgroups among 139 primary NF-pNETs

	ATRX-DAXX-loss-group	Isolated-Menin-loss-group	Unspecified-group	P-value
	(N = 42)	(N = 33)	(N = 64)	
Sex				0.062 #
Female	22 (52.4%)	10 (30.3%)	35 (54.7%)	
Male	20 (47.6%)	23 (69.7%)	29 (45.3%)	
Missing	0	0	0	
Age				0.37 §
Mean (SD)	59.5 (10.8)	61.2 (10.8)	56.9 (13.8)	
Median (IQR)	60.0 (53.2, 66.8)	63.5 (56.2, 68.0)	58.5 (51.0, 66.2)	
Min, Max	33.0, 81.0	37.0, 79.0	19.0, 78.0	
Missing	0	1	0	
Tumor size (cm**)**				< 0.001 §
Mean (SD)	5.0 (3.6)	2.3 (1.4)	3.0 (1.6)	
Median (IQR)	4.1 (2.5, 6.2)	2.0 (1.4, 2.7)	2.5 (1.9, 3.8)	
Min, Max	1.5, 18.0	1.0, 8.5	1.0, 7.0	
Missing	0	0	0	
Size, dichotomized (cm)				< 0.001 #
≤ 2	5 (11.9%)	20 (60.6%)	22 (34.4%)	
> 2	37 (88.1%)	13 (39.4%)	42 (65.6%)	
Missing	0	0	0	
Ki67				< 0.001 §
Mean (SD)	5.7 (4.8)	2.2 (1.9)	2.9 (3.1)	
Median (IQR)	4.8 (2.2, 7.7)	1.6 (1.0, 2.8)	1.8 (1.0, 3.6)	
Min, Max*	0.0, 20.0	0.0, 10.3	0.0, 15.0	
Missing	3	1	2	
WHO 2022 tumor grade				< 0.001 ¥
1	16 (38.1%)	26 (78.8%)	47 (73.4%)	
2	26 (61.9%)	7 (21.2%)	17 (26.6%)	
Missing	0	0	0	
WHO 2026 tumor grade				< 0.001 ¥
Grade 1	13 (33.3%)	25 (78.1%)	45 (72.6%)	
Grade 2a	20 (51.3%)	6 (18.8%)	15 (24.2%)	
Grade 2b	6 (15.4%)	1 (3.1%)	2 (3.2%)	
Missing	3	1	2	
Location				< 0.001 ¥
Corpus and tail	31 (73.8%)	30 (90.9%)	26 (41.3%)	
Head and uncinate	11 (26.2%)	3 (9.1%)	37 (58.7%)	
Missing	0	0	1	
ATRX/DAXX loss or ALT				< 0.001 ¥
Yes	42 (100.0%)	0 (0.0%)	0 (0.0%)	
No	0 (0.0%)	33 (100.0%)	64 (100.0%)	
Missing	0	0	0	
Menin				< 0.001 ¥
Loss	22 (57.9%)	33 (100.0%)	0 (0.0%)	
Preserved	16 (42.1%)	0 (0.0%)	64 (100.0%)	
Missing	4	0	0	
Lymphovascular invasion				0.0061 ¥
No	8 (27.6%)	19 (70.4%)	27 (52.9%)	
Yes	21 (72.4%)	8 (29.6%)	24 (47.1%)	
Missing	13	6	13	
Perineural invasion				0.13 ¥
No	10 (50.0%)	18 (78.3%)	23 (57.5%)	
Yes	10 (50.0%)	5 (21.7%)	17 (42.5%)	
Missing	22	10	24	
Regional node (pN) stage				0.039 ¥
N0	23 (60.5%)	20 (90.9%)	39 (70.9%)	
N1	15 (39.5%)	2 (9.1%)	16 (29.1%)	
Missing	4	11	9	
Primary tumor (pT) stage				< 0.001 ¥
1	3 (8.1%)	16 (51.6%)	19 (31.1%)	
2	16 (43.2%)	13 (41.9%)	28 (45.9%)	
3	18 (48.6%)	2 (6.5%)	14 (23.0%)	
Missing	5	2	3	
Disease recurrence				< 0.001 ¥
Non-recurred	22 (52.4%)	33 (100.0%)	56 (87.5%)	
Recurred	20 (47.6%)	0 (0.0%)	8 (12.5%)	
Follow-up (months)				0.58 §
Mean (SD)	40.5 (40.1)	41.3 (36.1)	46.9 (42.7)	
Median (IQR)	25.5 (12.0, 59.2)	26.0 (20.0, 55.0)	31.1 (18.0, 59.5)	
Min, Max	0.0, 193.0	1.0, 173.0	0.0, 195.0	
Missing	0	0	0	

# Chi-square test, ¥ Fisher-freeman-halton test, § Kruskal-wallis test, * numbers shown are rounded values

## Results

Description of overall cohort.

A total of 139 patients who underwent resection between 1993 and 2023 for a grade 1 or 2 sporadic NF-pNET were included for this study. The overall cohort had a nearly equal distribution of men and women (men, *n* = 72, 52%) with a median age of 61 years (range: 19–81 years) at the time of primary resection. The median tumor size was 2.6 cm (range: 1–18 cm), with 66% of tumors > 2 cm. Eighty-nine (64%) patients had WHO grade 1 tumor, while 50 (36%) patients had a WHO grade 2 tumor. ATRX loss and/or DAXX loss and/or ALT occurred in 30% (42/139) of patients and Menin loss in 40% (55/135) of patients. Consequently, 42 (30%) of patients were categorized into the ATRX-DAXX-loss-group, 33 (24%) into the isolated-Menin-loss-group, and 64 (46%) into the unspecified-group. 

### Description of Three Molecular Subgroups

Table 1 compares the differences between ATRX-DAXX-loss-group, the isolated-Menin-loss-group, and the unspecified-group, demonstrating that established prognostic factors, such as larger tumor size, higher grade, PNI, LVI, and pN1 stage, are more prevalent in the ATRX-DAXX-loss-group and the unspecified-group: Median tumor sizes were 4.10 cm (1.50–18.0), 2.00 cm (1.00–8.50), and 2.50 cm (1.00–7.00), respectively. When dichotomized at 2 cm, most tumors in the ATRX-DAXX-loss-group and the unspecified-group were > 2 cm (88% and 66% respectively), whereas in the isolated-Menin-loss-group the majority of tumors were ≤ 2 cm (61%). WHO grade 1 was more common in the isolated-Menin-loss-group and the unspecified-group (79% and 73% respectively), while grade 2 predominated in the ATRX-DAXX-loss-group (62%). Among grade 2 tumors, WHO grade 2b was most prevalent in the ATRX-DAXX-loss-group. LVI was present in 50% (53/107) of cases overall. It was reported more frequently in the ATRX-DAXX-loss-group (72%; 21 of 29 cases) than in the isolated-Menin-loss-group (30%; 8 of 27 cases). PNI was seen in 39% (32/83) of cases overall and was more frequently present in the ATRX-DAXX-loss-group (50%; 10/20 cases) and the unspecified-group (43%; 17/40 cases), compared to 22% (5/23) in the isolated-Menin-loss group. pN stage was predominantly N0, seen in 71% (82/115) of cases. pN1 stage was more frequent in the ATRX-DAXX-loss-group (39%) and the unspecified-group (29%) compared to isolated-Menin-loss-group (9%). 

### Prognostic Significance of Immunohistochemistry-Based Subgroups

In the overall cohort, the median follow-up was 28 months (range: 0–195 months) after primary resection during which disease recurred in 28 patients. 78% of patients had a follow-up of at least 1 year, and 25% were followed for more than 5 years. Nine patients had a follow-up of < 6 months (4 ATRX-DAXX-loss, 1 isolated-Menin-loss, and 4 unspecified), with one recurrence in the ATRX-DAXX-loss-group. Nineteen patients were followed for < 1 year (6 ATRX-DAXX-loss, 2 isolated-Menin-loss, and 11 unspecified), including four recurrences (two ATRX-DAXX-loss and two unspecified). Follow-up of < 2 years was available for 53 patients (20 ATRX-DAXX-loss, 11 isolated-Menin-loss, and 22 unspecified), among whom 12 recurrences occurred (three ATRX-DAXX-loss and nine unspecified).

**Fig. 2:**
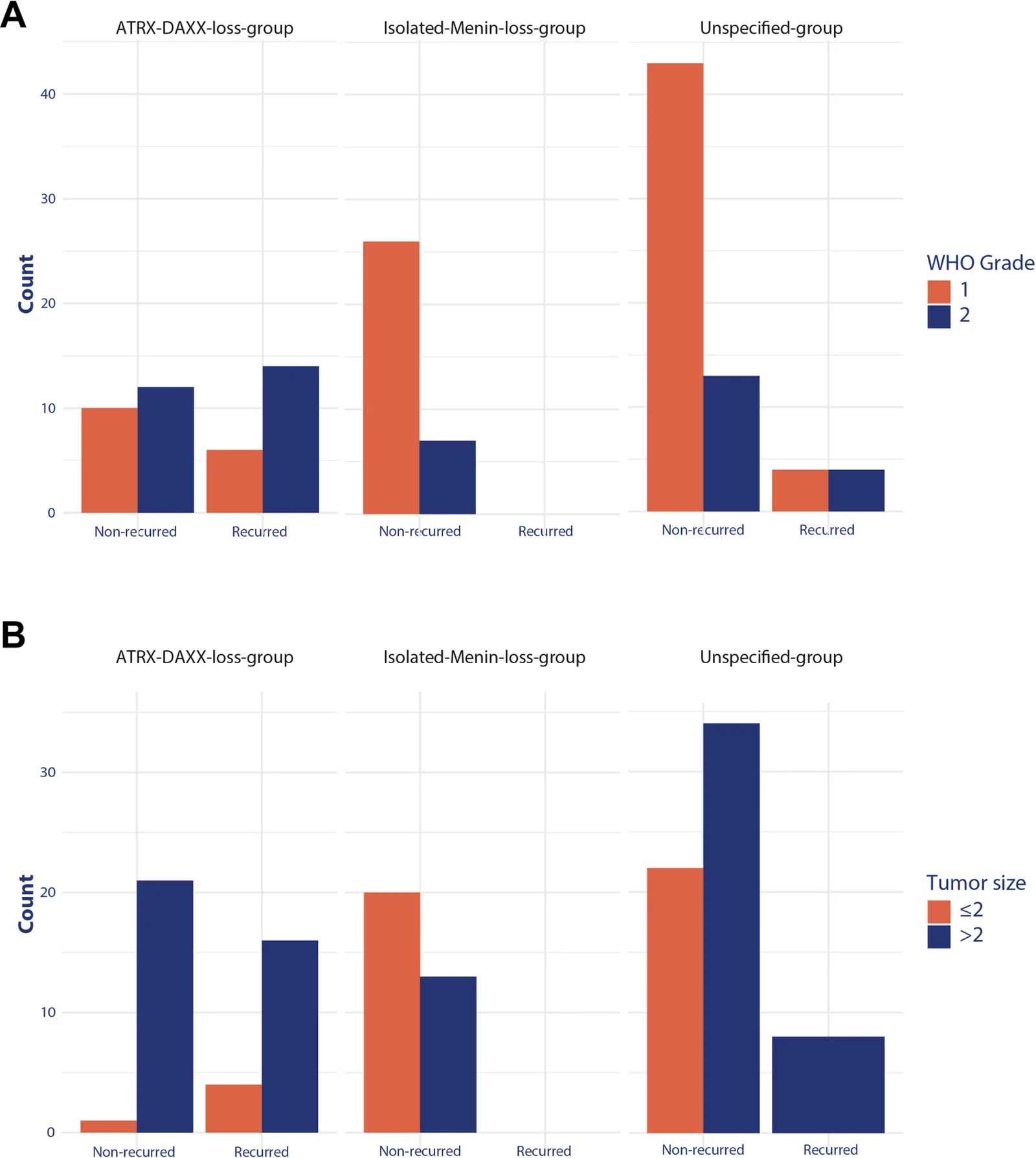
Tumor size and tumor grade in relation to recurred and non-recurred NF-pNETs in three different molecular subgroups. **A**) Tumor grade in relation to recurred and non-recurred NF-pNETs in three different molecular subgroups shows that recurred tumors in unspecified-group were both grade 1 and grade 2, **B**). Tumor size in relation to recurred and non-recurred NF-pNETs in three different molecular subgroups shows that all recurred tumors in unspecified-group were >2 cm tumors.

Interestingly, no disease recurrence was observed in the isolated-Menin-loss-group (Table 1), this subgroup included 13 tumors larger than 2 cm, seven WHO grade 2 tumors and two cases with regional lymph node metastasis. In contrast, tumors in the unspecified group were more frequently WHO grade 1; recurred tumors in this group were typically larger than 2 cm and were equally often classified as grade 1 and grade 2 (Fig. 2).

**Fig. 3:**
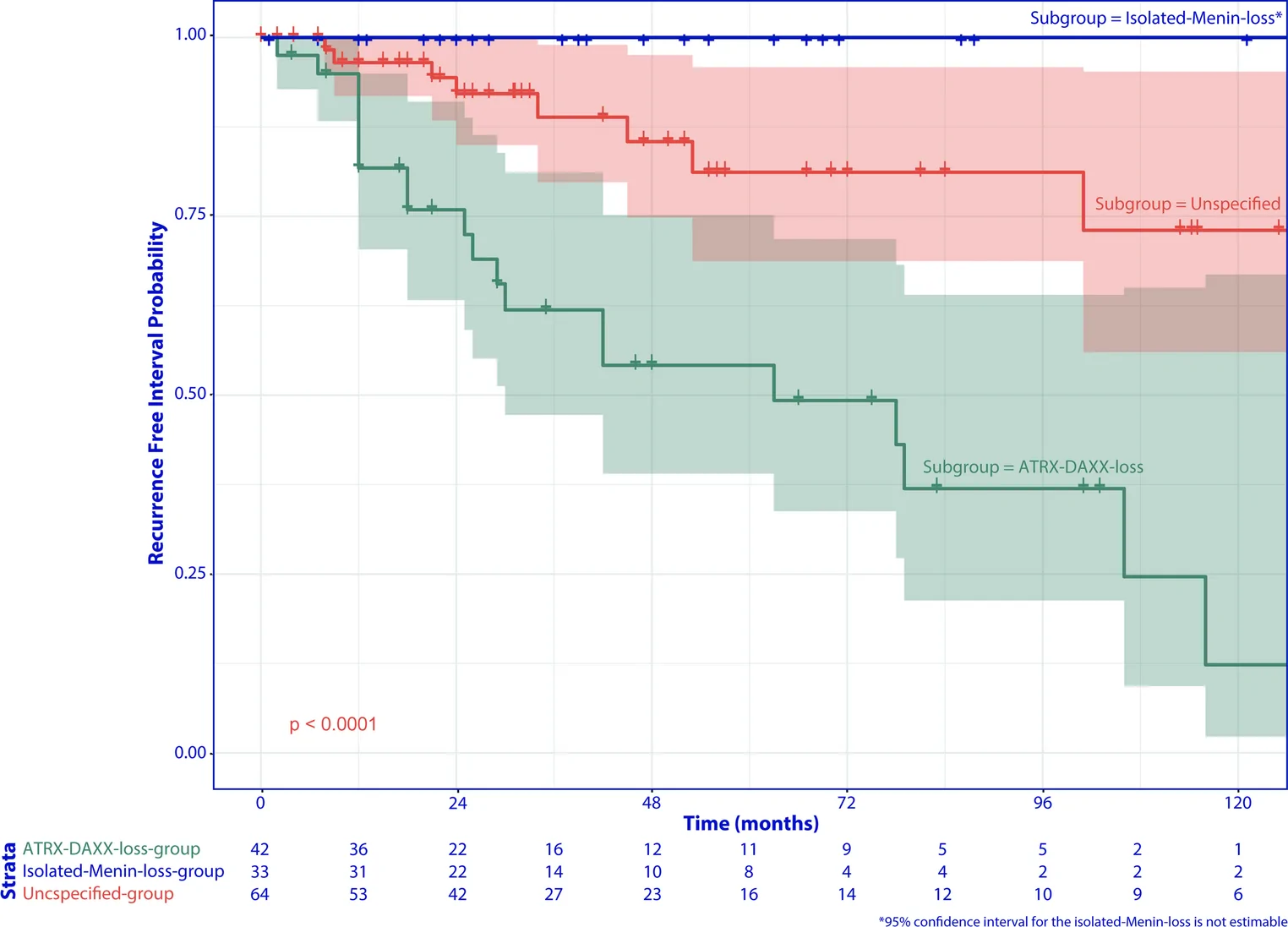
Kaplan Meier Curve comparing recurrence-free interval (RFI) after surgical resection for patients with NF-pNETs with three molecular subgroups. Log-rank test shows significant differences in RFI between three molecular subgroups.

Recurrence-free interval analysis (Fig. 3), showed statistically significant differences between the three subgroups (log-rank test *P*-value < 0.0001). The RFI of ATRX-DAXX-loss-group and unspecified-group was significantly decreased with 1-year, 5-year and 10-year RFI rates of 82% (95%CI: 70–95%), 54% (95%CI: 39–75%) and 12% (95%CI: 2–67%), and 97% (95%CI: 92–100%), 81% (95%CI: 69–96%) and 73% (95%CI: 56–95%) respectively. The patients at risk were 42, 11, 1 and 64, 16, 6 respectively. In contrast, 1-year, 5-year and 10-year RFI rates for isolated-Menin-loss-group were 100% (95%CI: 89–100%), 100% (95%CI: 66–100%) and 100% (95%CI: 29–100%) with patients at risk at those time points 33, 8 and 2 respectively.

Table 2 summarizes the results of the univariable and multivariable Cox regression models. In univariable analysis, the molecular subgroups were strongly associated with recurrence-free interval. Compared with the ATRX-DAXX-loss-group, patients in the isolated-Menin-loss-group had a substantially lower hazard of recurrence (HR 0.03, 95%CI: 0.00–0.55; *P* = 0.018), as did patients in the unspecified-group (HR 0.24, 95%CI: 0.10–0.54; *P* = < 0.001). After adjustment for the published prognostic model (included as its linear predictor) [10], the molecular subgroups lost statistical significance. However, the hazard ratios for recurrence-free interval changed little. The HR was 0.09 (95%CI: 0.01–1.63; *P* = 0.10) for the isolated-Menin-loss-group and 0.41 (95%CI: 0.17–1.00; *P* = 0.049) for the unspecified-group, both compared with the ATRX-DAXX-loss-group. Complete-case analysis reduced the sample size to 54% of the original cohort (*n* = 75). In this analysis, the isolated-Menin-loss subgroup remained associated with a lower recurrence risk in the univariable model (HR 0.04; 95% CI 0.00–0.29; *P* = 0.0002). After adjustment for the linear predictor, the effect estimate remained largely unchanged (HR 0.09; 95% CI 0.00–0.76, *P* = 0.023), consistent with the results of the imputed analysis. These findings suggest that the molecular subgroups may provide prognostic information beyond the established model.

**Table 2:** Association between molecular subgroups and recurrence-free interval among 139 primary NF-pNETs, before and after adjustment for grade, lymph node metastasis and perineural invasion∞ (Firth corrected Cox regression )

		Recurrence-free interval HR (95%CI)	
		Univariable analysis	Multivariable analysis
Molecular subgroups	ATRX-DAXX-loss-group	1 (ref.)	1 (ref.)
	Isolated-Menin-loss-group	0.03 (95%CI: 0.00–0.55; *P* = 0.018)	0.09 (95%CI: 0.01–1.63; *P* = 0.10)
	Unspecified-group	0.24 (95%CI: 0.10–0.54; *P* < 0.001)	0.41 (95%CI: 0.17–1.00; *P* = 0.049)

Abbreviations: *NF-pNETs *= non-functioning pancreatic neuroendocrine tumors, *HR *= hazard ratio, *CI *= confidence interval∞ Given the relatively small sample size available, we used the published prognostic model for recurrence in non-functioning pancreatic neuroendocrine tumors, based on tumor grade 2, lymph node metastasis, and perineural invasion as reported by Genç et al.[10] The published prognostic model was incorporated as a single linear predictor. Its estimated coefficient was 1.306 when fitted alone and 1.141 after inclusion of the molecular subgroups.

## Discussion

The clinical behavior of low-grade NF-pNETs is variable, and accurate and readily available prognostic markers are needed to tailor clinical post-operative management. In this study we subtyped WHO G1 and G2 NF-pNET in three molecular subgroups based on ATRX, DAXX and Menin immunohistochemistry. This revealed a favorable prognosis for patients with tumors that show immunohistochemical Menin loss and retained ATRX and DAXX expression, here referred to as isolated-Menin-loss-group, as compared to ATRX-DAXX-loss-group and the unspecified-group. These molecular subgroups provide supplementary prognostic information for RFI in addition to well-known prognostic factors tumor grade, regional lymph node status and PNI [10].

Furthermore, this study shows established prognostic factors such as tumor grade, PNI and lymph node metastases are associated with disease recurrence, but also emphasizes the ongoing need for novel biomarkers, as we observed disease recurrence even in small and low-grade tumors. The isolated-Menin-loss-group showed an indolent behavior, despite presence of WHO G2 and tumors > 2 cm in this group, highlighting the need for better prognostic biomarkers in addition to these conventional risk factors. Interestingly, the unspecified-group demonstrated a low recurrence rate of only 12.5% (8/64). Importantly, all tumors in this group that recurred were larger than 2 cm and predominantly showed other known adverse features, including LVI (5/6), PNI (4/5), and lymph node metastases (6/8). As this subgroup is defined by the absence of Menin, ATRX and DAXX loss alternative molecular driver alterations are likely involved in the pathogenesis of these tumors, highlighting the need to explore additional molecular prognostic factors in this subgroup specifically. Further refinement of prognostication may be achieved by unraveling the unspecified-group subgroup.

It should be noted that this study used a slightly different subgroup classification as compared to the index studies of Parilla et al. and Lawrence et al. [17, 18]. Our subgroups correspond to Parilla’s mpNET group 1 (our ATRX-DAXX-loss-group), mpNET group 2 (our isolated-Menin-loss-group) and mpNET group 3 (our unspecified-group). Whereas these studies distinguished mpNET groups 2 and 3 based on isolated chromosome 11 loss versus variable aneuploidy, our classification relied on loss versus retained Menin protein expression by immunohistochemistry. This distinction is important, as Parilla et al. observed that within the mpNET group 3 12% (11/90) of patients had *MEN1* mutations, but without isolated chromosome 11 loss and without *DAXX* or *ATRX* mutations, and those patients had a poorer overall and disease-specific survival than compared to mpNET group 1 [18]. With our immunohistochemistry-based classification, these patients may fall into the isolated-Menin-loss-group, which we consider the ‘favorable’ group. Although no recurrences were observed in the isolated-Menin-loss-group in our cohort, CNV analysis using whole exome sequencing could distinguish between true isolated chromosome 11 loss and a variable pattern of aneuploidies consistent with the unspecified-group in Parilla et al. [18].

The small number of patients and recurrences limited more extensive multivariable analyses. To estimate the independent effect as accurately as possible, we compared the molecular subgroups with a linear predictor composed of the most discriminative prognostic variables, as described by Genç et al. [10]. In this analysis, the isolated-Menin-loss-group no longer reached statistical significance and showed wide 95% confidence interval, likely due to the small number of patients and events, although the hazard ratio changed only modestly after adjustment (approximately 0.03 to 0.09). Relatively short follow-up further limited insight into long-term outcomes. Although the estimated 10-year RFI for the isolated-Menin-loss group was 100%, this estimate was based on only two patients remaining at risk at 10 years, and should therefore be interpreted with caution. Nevertheless, previous studies have shown that most recurrences occur within the first 3–5 years after surgery [7, 25]. We also note that there was a relatively high proportion of missing data in several relevant variables. To minimize the loss of statistical power and potential selection bias associated with a complete-case analysis, PNI and N-stage were imputed using the *smcfcs* package. However, as with any imputation approach, this method relies on assumptions regarding the missing data mechanism, and some degree of bias cannot be excluded. We further acknowledge the lack of centralized pathology review in this multi-institutional cohort spanning 30 years, which may have introduced inter-observer and temporal variability. In addition, lymphatic and vascular invasion were not assessed separately. Given the limited number of recurrence events, extensive multivariable analyses were not feasible, and these variables were not prioritized as they are not included in the linear predictor. Finally, resection margin status was not evaluated because of missing data, which may have influenced the observed recurrence rates. Larger cohorts with more recurrence events and longer follow-up are therefore needed to validate these findings, better define the incidence and recurrence risk of the indolent isolated-Menin-loss group, and determine the added prognostic value of molecular subgrouping alongside established well collected clinicopathological factors. Nevertheless, we hypothesize that biological and molecular characteristics, such as molecular subgroup classification, provide a more robust reflection of tumor behavior than conventional clinicopathological variables. The current study lays the groundwork for future prospective studies evaluating whether molecular risk stratification can be safely incorporated into clinical decision-making.

Over the past decade, many studies have shown that loss of ATRX, DAXX, and the presence of ALT are independent prognostic factors for NF-pNETs. Despite calls from clinicians to incorporate these findings into routine clinical practice [26, 27], they have been scarcely integrated and have had limited impact on clinical decision-making. While current established markers such as tumor size, tumor grade, LVI, PNI, and nodal involvement focus on identifying aggressive tumors, subtyping based on immunohistochemistry, mutation analysis, and CNV analysis as suggested by Parilla et al. and Lawrence et al. [17, 18], offers an opportunity to identify indolent tumors. In the present study, we demonstrate that similar prognostic subgroups can be identified using immunohistochemistry alone, which is a method that is both accessible and easily implementable in daily surgical pathology practice. Importantly, this approach could help address two key clinical dilemmas. First, since all pNETs currently undergo lifelong postoperative follow-up regardless of prognosis, identifying a subgroup with an indolent profile may justify a reduced follow-up strategy, which in turn could improve quality of life and reduce health care costs. Second, relying on molecular subtyping, could increase confidence in managing small NF-pNETs with active surveillance protocols. Looking ahead, these findings may even support reconsidering the current 2-cm threshold used in patient management, as larger tumors with indolent characteristics might also benefit from surveillance strategies, potentially sparing patients the risks and complications associated with pancreatic surgery.

In summary, we observed three distinct prognostic groups based on the immunohistochemical expression of ATRX, DAXX, and Menin. As expected, ATRX or DAXX loss was consistently associated with poorer outcomes. Importantly, we identified a low-risk group characterized by isolated-Menin-loss without disease recurrence. As this assay relies on routine, widely available immunohistochemistry, it is immediately applicable in clinical practice and has the potential to directly facilitate risk-adapted follow-up strategies. 

*The recurrence free interval probabilities with 95% confidence intervals for the isolated-Menin-loss-group were calculated using the *bpcp* package and were 100% (95%CI: 89–100%), 100% (95%CI: 66–100%) and 100% (95%CI: 29–100%) at 1, 5 and 10 years respectively.

## Data Availability

All data supporting the findings of this study are available upon reasonable request.

## Abbreviations

ALT: Alternative lengthening of telomeres
AMC: Asan Medical Center
ATRX: Alpha-Thalassemia/X-linked mental Retardation
AUMC: Amsterdam University Medical Centers
CNVs: Copy number variations
DAXX: Death Domain Associated Protein
FISH: Fluorescence in situ hybridization
G1: WHO grade 1
G2: WHO grade 2
HR: Hazard ratio
LVI: Lymphovascular invasion
MEN1: Multiple endocrine neoplasia type 1
NF1: Neurofibromatosis type 1
NF-pNET: Non-functioning pancreatic neuroendocrine tumors
pNET: Pancreatic neuroendocrine tumors
PNI: Perineural invasion
RFI: Recurrence-free interval
SST-PET/CT: Somatostatin receptor Positron Emission tomography/Computed Tomography
TMA: Tissue Micro Array
TSC: Tuberous sclerosis complex
UMCU: University Medical Center Utrecht
UPMC: University of Pittsburgh Medical Center
VHL: Von Hippel-Lindau
WHO: World Health Organization
